# Modeling Viral Evolutionary Dynamics after Telaprevir-Based Treatment

**DOI:** 10.1371/journal.pcbi.1003772

**Published:** 2014-08-07

**Authors:** Eric L. Haseltine, Sandra De Meyer, Inge Dierynck, Doug J. Bartels, Anne Ghys, Andrew Davis, Eileen Z. Zhang, Ann M. Tigges, Joan Spanks, Gaston Picchio, Tara L. Kieffer, James C. Sullivan

**Affiliations:** 1 Vertex Pharmaceuticals Incorporated, Boston, Massachussets, United States of America; 2 Janssen Infectious Diseases BVBA, Beerse, Belgium; 3 Janssen Research & Development, Titusville, New Jersey, United States of America; University of California San Diego, United States of America

## Abstract

For patients infected with hepatitis C virus (HCV), the combination of the direct-acting antiviral agent telaprevir, pegylated-interferon alfa (Peg-IFN), and ribavirin (RBV) significantly increases the chances of sustained virologic response (SVR) over treatment with Peg-IFN and RBV alone. If patients do not achieve SVR with telaprevir-based treatment, their viral population is often significantly enriched with telaprevir-resistant variants at the end of treatment. We sought to quantify the evolutionary dynamics of these post-treatment resistant variant populations. Previous estimates of these dynamics were limited by analyzing only population sequence data (20% sensitivity, qualitative resistance information) from 388 patients enrolled in Phase 3 clinical studies. Here we add clonal sequence analysis (5% sensitivity, quantitative) for a subset of these patients. We developed a computational model which integrates both the qualitative and quantitative sequence data, and which forms a framework for future analyses of drug resistance. The model was qualified by showing that deep-sequence data (1% sensitivity) from a subset of these patients are consistent with model predictions. When determining the median time for viral populations to revert to 20% resistance in these patients, the model predicts 8.3 (95% CI: 7.6, 8.4) months versus 10.7 (9.9, 12.8) months estimated using solely population sequence data for genotype 1a, and 1.0 (0.0, 1.4) months versus 0.9 (0.0, 2.7) months for genotype 1b. For each individual patient, the time to revert to 20% resistance predicted by the model was typically comparable to or faster than that estimated using solely population sequence data. Furthermore, the model predicts a median of 11.0 and 2.1 months after treatment failure for viral populations to revert to 99% wild-type in patients with HCV genotypes 1a or 1b, respectively. Our modeling approach provides a framework for projecting accurate, quantitative assessment of HCV resistance dynamics from a data set consisting of largely qualitative information.

## Author Summary

Hepatitis C virus (HCV) chronically infects approximately 170 million people worldwide. The goal of HCV treatment is viral eradication (sustained virologic response; SVR). Telaprevir directly inhibits viral replication by inhibiting the HCV protease, leading to high SVR rates when combined with pegylated-interferon alfa and ribavirin. Telaprevir-resistant variants may be detected in the subset of patients who do not achieve SVR with telaprevir. While the clinical impact of viral resistance is unknown, typically the telaprevir-sensitive virus re-emerges after the end of treatment due to competition between the telaprevir-sensitive and resistant variants. Previous estimates of these competition dynamics were obtained from population sequence data, which are qualitative and have a limited sensitivity of ∼20%. We sought to improve these estimates by combining these data with clonal sequence data, which are quantitative and have a sensitivity of ∼5%, and using quantitative modeling. The resulting model, which was verified with an independent data set, predicted that the median time for telaprevir-resistant variants to decline to less than 1% of the viral population was ≤1 year. Our modeling approach provides a framework for accurately projecting HCV resistance dynamics from a dataset consisting of largely qualitative information.

## Introduction

Hepatitis C is an inflammatory infection of the liver caused by the hepatitis C virus (HCV). HCV chronically infects approximately 170 million people worldwide [1]. HCV infection is a major risk factor for cirrhosis and hepatocellular carcinoma, and has become one of the leading causes of both liver transplant and cancer-related death in the United States [2], [3]. In contrast to other chronic viral diseases such as HIV and HBV, the goal of HCV treatment is eradication of the virus as determined by achievement of a sustained virologic response (SVR).

Telaprevir is a direct-acting antiviral that inhibits viral replication by binding to the active site of the HCV NS3-4a protease, an enzyme essential for viral replication [4]–[6]. In combination with pegylated-interferon alfa (Peg-IFN) and ribavirin (RBV), telaprevir increased SVR rates over Peg-IFN/RBV alone [7], [8]. Telaprevir exerts a strong directional selective pressure on the viral population, which leads to enrichment of variants with decreased sensitivity to the inhibitor. These telaprevir-selected variants have been well characterized and occur at or near the catalytic site of the protease, resulting in decreased sensitivity to telaprevir and other HCV protease inhibitors [9]. Given that other protease inhibitors besides telaprevir may be included as components of future drug regimens for patients that fail a telaprevir-based regimen, presence of telaprevir resistant variants may limit future treatment options. It is therefore essential to understand viral evolutionary processes and the rates at which telaprevir-resistant variants are outcompeted by wild-type virus to optimally inform patient's future treatment options.

In clinical studies, telaprevir-resistant variants were identified in the majority of patients who did not achieve an SVR with telaprevir treatment [10]–[13]. Monitoring of telaprevir-resistant variants after treatment failure revealed that these variants tend to be replaced over time by telaprevir sensitive, wild-type (WT) virus [14], presumably due to the lower intrinsic fitness of the resistant variants [10], [11]. However, monitoring was performed only by direct sequencing of RT-PCR products amplified from HCV RNA extracted from patient plasma (*i.e.*, ‘population sequencing’). Population sequencing infers genetic variation within a population from polymorphic peaks within sequence chromatograms, and therefore provides only qualitative information about the frequency of the variants. Industry-standard interpretation of these data assumes a limit of sensitivity of minority variant detection of ∼20% (see, for example, [12], [15]–[18]). Thus, although these clinical studies analyzed a large number of samples [14], the interpretation of the results is limited by the sensitivity of the assay used.

The analysis reported here builds upon the work of Sullivan et al. [14] in two key ways. First, the data set was augmented with clonal sequence data from a subset of the patients previously analyzed. The previous work used only population sequence analysis. Compared to population sequence analysis, clonal sequence analysis has a LOD of ∼5% and provides a quantitative estimate of the frequency of resistant variants in a patient sample. Second, computational modeling was used to integrate the qualitative information provided by population sequence data with the quantitative information provided by clonal sequence data. Overall, the combination of these two approaches resulted in a more quantitative and patient-level understanding of resistant variant evolution which allows extrapolation of viral resistance quantification both beyond the sampling period and to a greater sensitivity than can be estimated by the bioanalytical methods alone.

In Sullivan *et al.* [14], we performed Kaplan-Meier analysis across a population of patients to determine the ‘time-to-wildtype’. ‘Wildtype’ (WT) simply indicated that resistance was present at 20% or less of the viral population. From both a scientific and clinical standpoint, this answer is unsatisfactory: 19% of the viral population could be resistant by population sequencing and represent a major undetectable reservoir of drug resistance. Our modeling here addresses this significant shortcoming by allowing calculation of time-to-event analyses across patients based on levels of resistance below the sequencing assay limits of detection (e.g., 1%). Additionally, in our present analysis, we model rates of viral decline within individual patients. This ‘within patient’ analysis allows calculation within an individual of the rate of decline of resistant virus, and therefore allows (1) extrapolation of resistance levels at time points which were not sampled and (2) calculation of the time it takes for a given patient to revert to a given % resistance (*e.g.*, 1%). Significantly, population level analyses can be performed across patients for any of these metrics. As such, our models and pipeline described here form a framework for future antiviral resistance monitoring programs. We propose that this methodology could be applied across both antiviral and antibacterial therapeutics.

## Results

### Modeling Context

**Figure 1:**
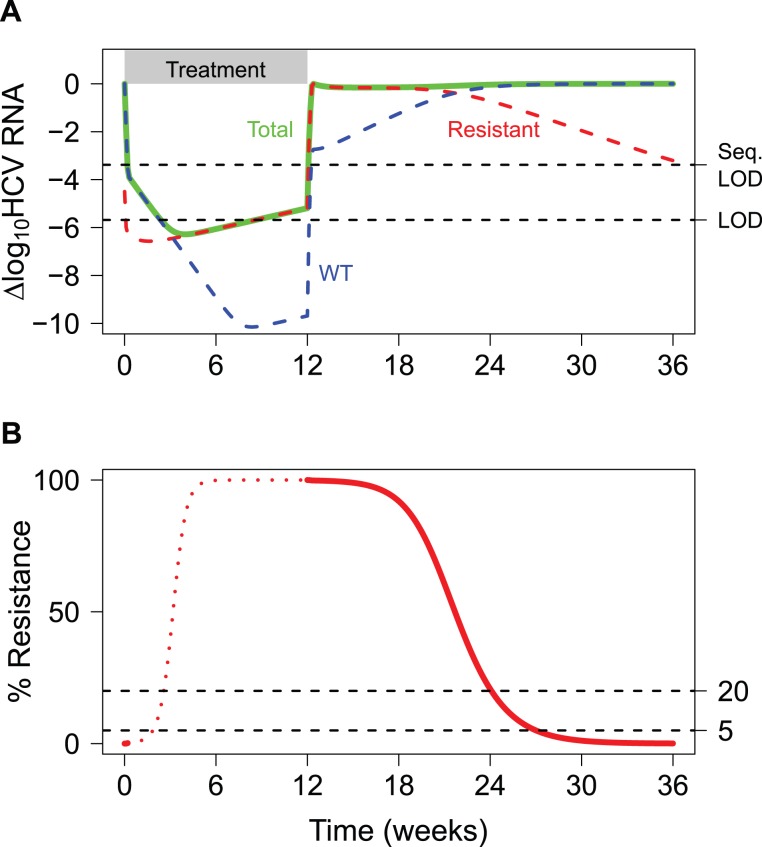
Hypothetical viral dynamics for a patient with viral breakthrough during telaprevir-based treatment. (A) Dynamics for the total viral load (Total, green), wild-type virus (WT, blue), and a telaprevir-resistant variant (Resistant, red) during and after treatment with telaprevir-based treatment. LOD is the limit of detection for the ‘total’ viral load quantification, and Seq. LOD is the limit of detection above which sequencing can be reliably performed (1000 IU/ml). The treatment phase is shown by the gray bar. (B) Corresponding percent resistance dynamics on a linear scale. Viral sequencing can be performed when the total viral load exceeds the sequencing assay LOD (solid red curve). The dashed lines at 20% and 5% show the limits of detection for population and clonal sequence data, respectively.

To illustrate the concept of resistance monitoring, Figure 1 displays hypothetical viral dynamics for a patient who experiences viral breakthrough before Week 12 of treatment. Up to Week 12, the WT virus is strongly suppressed by the treatment, whereas some resistant variants may be able to replicate even in the presence of the treatment. At Week 12, all treatment is terminated, and the competition between WT and resistant virus continues in the absence of drug selective pressure. By Week 24, the WT virus overtakes the resistant variant population as the dominant viral species, and the level of the resistant variant continues to decline (Figure 1A). Resistance monitoring in patients with HCV infection can effectively quantify the frequency of resistant virus over time, when the total viral load exceeds the sequencing limit of detection (Seq. LOD; see Figure 1). In the example shown, sequence analysis can be performed at the beginning of treatment and after treatment has stopped, as denoted by the solid red line. This analysis is focused on the latter portion of this time frame, once treatment is terminated. Dynamic sequence data from 388 genotype 1 HCV patients who did not achieve an SVR with a telaprevir-based regimen were available for the analysis. Figure S1 presents more detailed information on the number of population and clonal sequence data points obtained per patient.

### Fitting the Model with Population and Clonal Sequence Data

**Figure 2:**
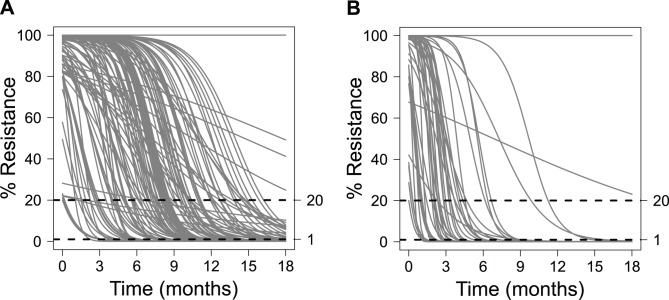
Model fits for resistant variant decline. The fitted dynamics of resistant variant loss for each patient are shown for (A) genotype 1a and (B) genotype 1b.

The logistic model given by Equation 3 was used to describe the dynamics of the resistant virus for each individual patient. The model assumed that the treatment-free equilibrium level of resistance is 0%. To fit this model to the available data, a two-step estimation procedure was used. First, we fit the model to the subset of patients who had both clonal (quantitative) and population (qualitative) sequence data after treatment failure. For model fitting, each qualitative (population sequence) data point was converted into a ranged value, with binned resistance values of (1) 0 to 20% (*i.e.*, WT population result), (2) 20 to 80% (*i.e.*, polymorphic population sequence result), or (3) 80 to 100% (*i.e.*, resistant population result). HCV genotypes 1a and 1b were fit independently because their resistance profiles are different [14]. We used this fitted subset to approximate the expected prior parameter distributions for each genotype using a log-normal distribution. We then refit all patients individually, including those who had clonal sequence data (*i.e.*, those that had already been fit), approximating the estimated parameters as distributed according to these priors. The resulting fits for all patients are shown in Figure 2. For patients who had only population sequence (qualitative) data, imposing this prior allowed us to determine a unique set of parameters for those individual patients that fit the data.

**Figure 3:**
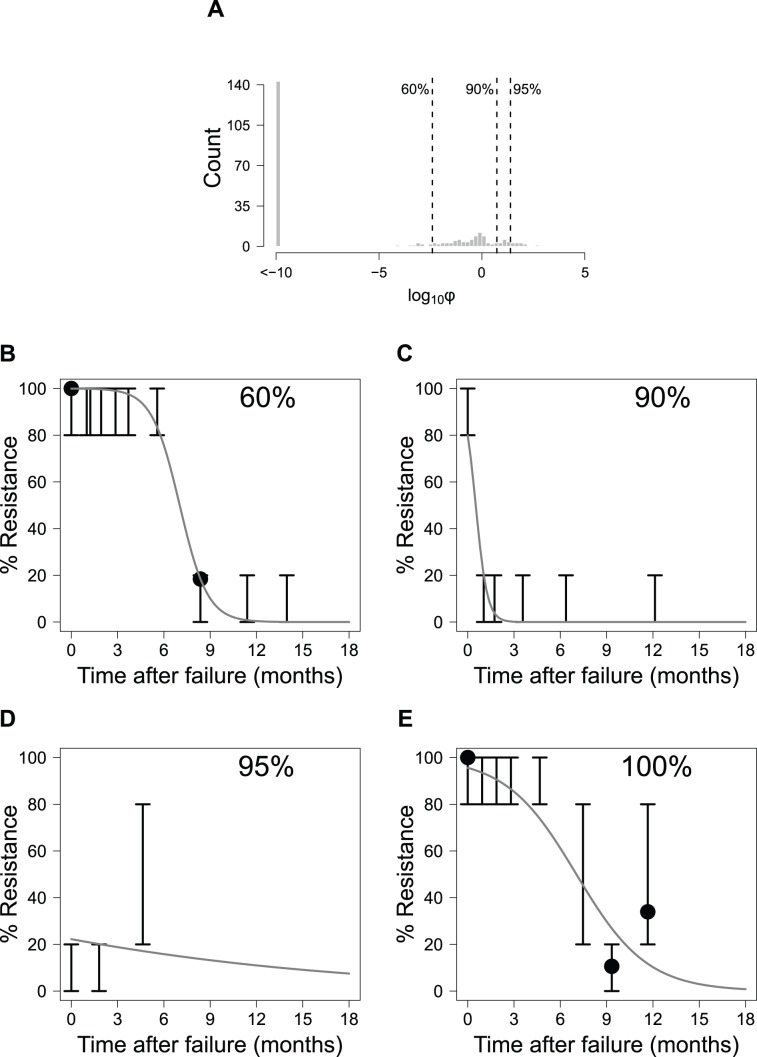
Model fit for patients with genotype 1a HCV. (A): Histogram of the log_10_ objective function values (φ; see Equation 5) for all patients with genotype 1a. Dashed lines and numbers show quantile information for the fits. Also shown are representative fits for patients whose objective function values fall in the (B) 60%, (C) 90%, (D) 95%, and (E) 100% quantiles. Solid lines represent model predictions, solid points represent the clonal sequence data, and error bars show the range for population sequence results.

**Figure 4:**
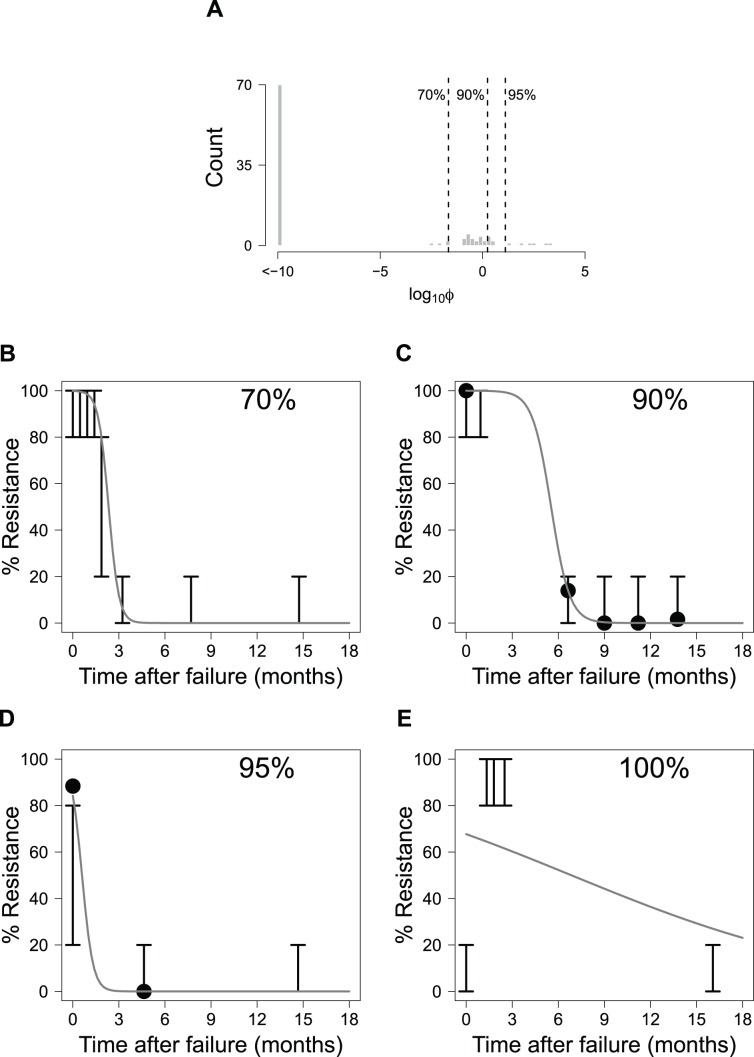
Model fit for patients with genotype 1b HCV. (A): Histogram of the log_10_ objective function values (φ; see Equation 5) for all patients with genotype 1b. Dashed lines and numbers show quantile information for the fits. Also shown are representative fits for patients whose objective function values fall in the (B) 70%, (C) 90%, (D) 95%, and (E) 100% quantiles. Solid lines represent the model predictions, solid points represent the clonal sequence data, and error bars show the range for population sequence results.

To examine how well the model fit the data across all patients, histograms of the objective function values (φ) were generated (Figure 3(A) and Figure 4(A)). Each φ value represents a scalar quantity that can be used to assess how well the model fits the result for a single patient, with larger φ values signifying worse fits to the data. In Figure 3 (B–E) and Figure 4 (B–E), representative fits for individual patients are shown for various quantiles to demonstrate the quality of fit for different φ values.

### Assessing the Model Using Deep Sequence Analysis

**Figure 5:**
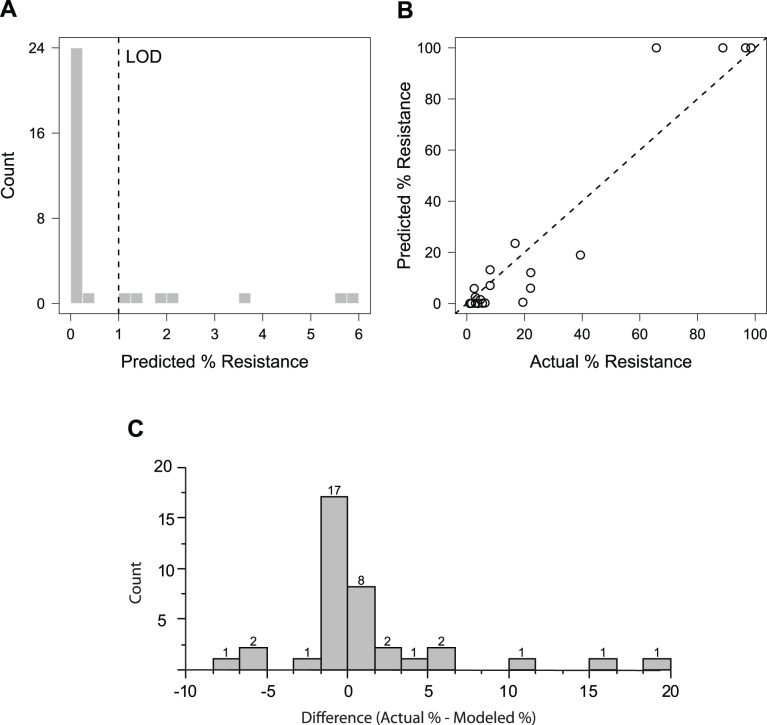
Validation of the model predictions using deep sequence data. Comparison of the model-predicted and actual (deep sequence) resistance frequency. (A) Predicted resistance frequency for samples with undetectable resistance by deep sequencing. (B) Predicted versus actual resistance frequency for samples with measurable resistance by deep sequencing. The p-value of the prediction is <10^−4^ as determined by Monte Carlo simulation. (C) The differences between the modeled and actual resistance frequency are depicted in the histogram (counts indicated above each bar), with a median % difference of -3.3e-5 and a mean % difference of 1.3.

To assess the predictive capability of the model, we compared model predictions to resistance quantified by massively parallel sequencing (deep sequencing; DS) from a subset of samples included in our modelling analysis using an Illumina platform (LOD: 1%). These data provided a more quantitative measure of resistance as resistance from these samples had previously been determined using only population sequencing. As the patients and time points chosen for quantification were selected and analyzed independently of this modeling work [19], they served as an independent validation of our approach. Because we fit our model (Equation 3) to each individual patient, we were able to predict, for each patient with DS data, the expected resistance at the precise time of the DS. There were 52 time points sequenced, each from a different patient. 32 samples had no detectable resistant virus (below the LOD), and the remaining 20 samples had 1.06%–98.58% resistant virus. For samples with no detectable resistance, the model predicted that the majority (n = 25; 78%) should have resistance values below 1% (Figure 5(A)). For all samples sequenced, the model predictions showed good agreement with the sequence results (Figure 5(B)). To determine the significance of these results, Monte Carlo simulation was used to generate 10^4^ equivalently sized datasets for the DS samples. None of these datasets had a smaller sum of squared errors than that observed in this analysis, indicating that our observations have a <10^−4^ probability of being generated by chance alone. Additionally, we assessed the null hypothesis that the difference between the actual and predicted % resistance values was equal to 0 (Figure 5(C)). After arcsine square root transformation of the differences, neither a t-test (*p* = 0.86) nor a Wilcoxon-signed rank test (*p* = 0.16) suggest a significant difference between the actual and model-predicted results.

### Model Predictions: Reversion from Resistant to WT Virus

**Figure 6:**
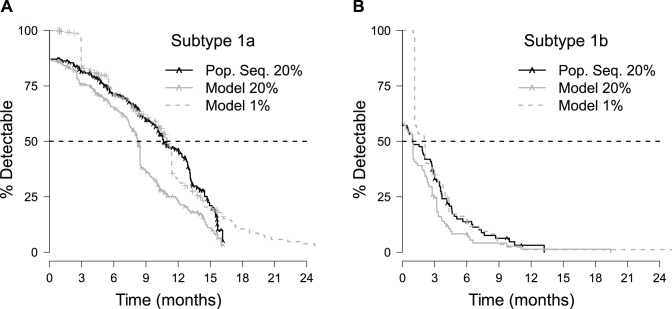
Kaplan-Meier curves for time-to-20% determined by population sequencing, model-predicted time-to-20%, and model-predicted time-to-1%. Results for patients with HCV subtypes 1a and 1b are shown in plots (A) and (B), respectively. Hash marks (∧) denote the censored observations indicating the time of the last visit for patients with virus that did not revert to <20% resistant. For clarity, these patients are explicitly denoted on the population sequence (“Pop. Seq.”) and model-predicted time-to-20% resistance curves only.

The model was used to predict population statistics for reversion of virus from the resistant to the WT, non-resistant state. Specifically, Kaplan-Meier analysis was used to calculate the median time to reversion for each HCV subtype. Previously, the estimated time it takes for a population of patients to revert from resistant to WT virus was calculated using only population sequence data [14]. Given the 20% sensitivity of that sequence method, this time was considered to represent the ‘time-to-20%.’ Here, those results were compared against the model derived time-to-20% estimates. The modeled Kaplan-Meier analysis of this reversion was less than (genotype 1a) or equal to (genotype 1b) the time-to-20% estimated by population sequence data (Figure 6, Table 1). For both genotypes, the upper 95% confidence interval for the median model prediction is lower than that for the population sequence result.

**Table 1:** Population sequence-based and model-predicted median reversion times.

	Population Sequence		Model Prediction		Model Prediction	
HCV subtype	Time-to-20%, months (95% CI)	Censored/Total (n/N)	Time-to-20%, months (95% CI^1^)	Censored/Total (n/N)	Time-to-1%, months (95% CI^1^)	Censored/Total (n/N)
**1a**	10.7 (9.9,12.8)	114/255	8.3 (7.6,8.4)	75/255	11.0 (10.3,11.4)	75/255
**1b**	0.9 (0.0,2.7)	9/105	1.0 (0.0,1.4)	5/105	2.1 (1.1,2.6)	5/105

1 The 95% CI assumes a single value for each patient and does not incorporate uncertainity of individual predictions (see Text S1).

**Figure 7:**
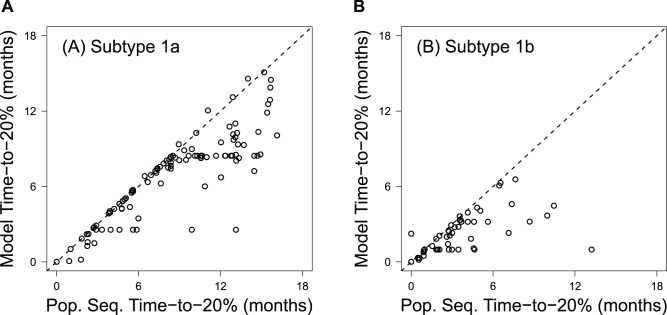
Comparison of the population sequence- and model-predicted time-to-20% for HCV subtypes 1a (A) and 1b (B). The X-axis represents the inferred time-to-loss of detectable resistance by population sequencing and reflects the first visit wherein the patient did not have detectable resistant variants. The Y-axis relies on the algorithms defined here, wherein the rate of loss is modeled continuously for each patient. The majority of the data points fall to the right of the unity line, indicating that the model predicts more rapid times-to-20% than those estimated from population sequence data.

Notably, fewer patients are censored in the modeled results as compared with the population sequence results (Table 1). This difference results from patients whose last population sequence data point is polymorphic (i.e., between 20% and 80% resistant). Because resistance is still present, the direct population sequence-based Kaplan-Meier survival analysis censors these points. In contrast, the model can predict reversion times for these patients. For patients that are considered to have achieved ≤20% resistance by the model and population sequence data (*i.e.*, patients whose last sequenced time point had no detectable resistance by population sequence data), the model consistently predicts shorter times-to-20% resistance than the population sequence results (Figure 7).

The predicted time-to-1% resistance was also determined as this value represents the measure of resistance theoretically obtainable by recent massively parallel sequencing approaches (e.g. [19]). Interestingly, these predictions are similar to the estimated time-to-20% resistance determined using population sequence data alone (Table 1, Figure 6). Note that these predictions did not account for uncertainty in the parameter estimates for individual patients. Monte Carlo simulations of this uncertainty suggested that it minimally affected the median reversion time determined by Kaplan-Meier analysis (see Text S1).

The predicted time-to-1% resistance was also used to assess the effect of a number of covariates on reversion times. The covariates assessed were: (1) baseline resistance status, (2) failure modality, (3) prior treatment status, and (4) the length of time PR treatment persisted after the time of treatment failure (Figure S2). Of these covariates, the presence of resistance at baseline exerted a large effect on resistant variant retention after treatment failure which was statistically significant in the case of genotype 1a infections. The data suggest that resistance retention rates are greater for patients that already had resistance variants present at baseline prior to treatment. Similarly, the data suggest that genotype 1a infected patients who experience on treatment virologic failure (as compared to patients that relapse after the end of treatment) as a group have statistically significantly longer retention times of resistant variants. Significantly, by protocol, a subset of patients that experienced on treatment virologic failure continued to be treated with pegylated interferon and ribavirin (PR) after they experienced viral breakthrough. Consequently, Cox proportional hazards analysis of this covariate is largely overlapping with the analysis of failure modality and suggests that continued dosing with PR after treatment failure also increases the time that it takes for a viral population to revert to 1% resistant. In contrast to these two covariates, prior treatment status did not provide any strong signals for affecting the retention time of resistant variants.

## Discussion

Previously, resistance monitoring of patients who did not achieve an SVR with telaprevir-based treatment in the Phase 3 studies ADVANCE, REALIZE, and ILLUMINATE showed that, after treatment failure in the absence of drug, resistant variants decline over time and are replaced by WT (drug-sensitive) virus [14]. One limitation of this previous work is that the analysis included only qualitative data obtained from population sequencing [14]. To provide a more quantitative understanding of viral dynamics after telaprevir-based treatment, we generated a quantitative clonal sequence dataset for a subset of the patient samples. We then employed mathematical modeling to continuously fit both the quantitative (clonal sequence) and qualitative (population sequence) datasets. The model explored the rate at which resistant virus reverts to WT virus (see Figure 1).

Of note, existing methodology for analysis of population sequence results allows estimation of resistance levels at only discrete time points, and therein can only describe resistance levels in gross bins (*e.g.*, 0–20%; 20–80%, or 80–100%). By developing patient-level models that describe how resistance levels change over time, and by merging population and clonal sequence data, our analysis provides substantial additional advantages:

1. Because the model uses a continuous function, a patient's resistance level can be calculated within a patient's population sequence binned result at a given time (*e.g.*, a 20–80% population sequence result could be calculated, as, for example, 20%).
2. In contrast to analysis of individual data points (as in Sullivan *et al.* [14]), our model allows estimation of resistance levels at points in time for a given patient that have not been sampled.
3. Both population and clonal sequencing have limitations in terms of their sensitivity, namely 20% and 5% resistance, respectively, as they are commonly employed. Our model allows for estimation of resistance levels below the sensitivity of the two data types used to generate the models.

### Model Performance

Overall, the results suggest that the model captures the resistance dynamics for the majority of patients quite well (Figure 3 and Figure 4). We found that numerous patients with the best model fits (φ≤10^−10^) had only population sequence data. We observed that the resistance dynamics for these patients were well fit by the population average parameters. Of note, one of the reasons why these patients' dynamics were well fit may be explained by the lack of specific information within the population sequence dataset since data are binned into ranges of between 0 and 20%, 20 and 80%, and 80 and 100% resistance. As such, many fits are possible through many of the population sequence curves which are consistent with the observed population sequence results.

Figure S3 illustrates this point as substantial variability in the model fits is observed in the two patients (A and B) having only population sequence data, whereas the variability is notably diminished in the patient C having both population and clonal sequence data.

In order to validate the model, we compared the quantitative model predictions of % resistance against actual quantitative results generated by an independent test set (DS). Monte Carlo simulation analyses suggest that the model predictions of % resistance for this test set are accurate with the null hypothesis of equivalence between the methods not refuted. The consistency between the model predictions and the DS results (1% resistance sensitivity) suggests that the model can accurately predict the resistance dynamics between 100% and 1% resistance even though neither of the sequence data types (population and clonal) used to train the model had sensitivities below 5%.

### Model Limitations

The model cannot fit two modalities of viral evolution. First, the model cannot fit patients whose % resistance increases over time because the model's logistic expression decreases monotonically over time. For example, as in the rare case shown in Figure 4(E), the measured resistance dynamics start at 0–20% resistance, change to 80–100% resistance, and then revert again to 0–20% resistance. Not surprisingly, this patient had the worst fit viral dynamics for the genotype 1b population. Such phenomena were observed infrequently, and are likely attributable to the stochasticity associated with PCR amplification in populations with HCV RNA levels near the assay LOD.

Second, the model does not accurately fit virus that appears to have a natural resistance level greater than 1% (e.g., Figure 3(E)). While an equilibrium resistance level of ∼20% would result in a better fit of the data from this patient, the final equilibrium resistance was fixed at 0% for all patients. Due to the dynamic nature of viral evolution after the strong selective pressure of the direct-acting antiviral is removed and the potential stochasticity of PCR amplification, the longitudinal sampling for this patient may not have been sufficiently long to capture the dynamics implied by the model's functional formula. Support for the placement of equilibrium resistance levels below 20% and closer to 0% is provided by a previous analysis by Bartels et al., who found that none of 3447 patients assayed for resistance by population sequencing had naturally occurring telaprevir resistant variants present as polymorphisms [18].

### Comparison to Existing Dynamic Models of HCV Resistance

To the best of our knowledge, population sequence data have not been explicitly used in any mechanistic modeling analyses thus far. Previously, clonal sequence data were used to construct a multi-variant HCV dynamic model that explained the dynamics of specific telaprevir-resistant variants before and after telaprevir treatment [20], [21]. This model was originally developed by fitting viral kinetics from patients treated with telaprevir monotherapy, with clonal sequence data used to quantify the relative fitness of specific telaprevir-resistant mutants [20]. The model was then refined in order to predict SVR rates by estimating relative fitness rates of different resistant variants using viral kinetics from Phase 2 telaprevir studies, but in this refinement no additional sequence data beyond the Phase 1 clonal sequence data were used to estimate model parameters [21]. Similarly, Rong et al. used an HCV model accounting for drug-resistant and drug-sensitive viruses to explain viral dynamics with telaprevir-based treatment; only clonal sequence data were used to inform the model parameters [22]. These modeling works differ from ours in two primary ways. First, these prior models [20]–[22] used clonal sequence data from a Phase 1 study with small numbers of patients (i.e., tens). In contrast, our work used both clonal and population sequence data from Phase 3 studies with large numbers of patients (i.e., hundreds). Second, these prior models [20]–[22] included substantially more mechanistic detail and greater numbers of free parameters than the approach presented here. However, Ganusov et al. elegantly demonstrated that these more complex models reduce to the simpler model used here under the limiting assumptions of a variant initially present at low frequency (in this case, WT) and a small mutation rate [23]. Thus, both the current analysis and the aforementioned analyses implicitly employ the same basic framework. Namely, viral dynamics are characterized as a competition between viral variants with different fitness levels, and the dynamics of the variant most fit for the environment (e.g., with versus without treatment) outcompeting the other variants are described by exponential growth capped at a specific maximum. The relative simplicity of the approach presented here (only two free parameters per patient) provides a more tractable framework for addressing the quantitative questions surrounding resistance reversion and is less likely to be subject to model over-fitting.

### Model Predictions

We used Kaplan-Meier estimation to determine the expected time frames for given events (e.g., the time-to-20% reversion, time-to-1% reversion). This data rich Kaplan Meier analysis (Figure 6) used all available data, and included 391 distinct visit results (Figure S1). We found that population sequencing provides a conservative estimate for the time-to-20% reversion (Figure 6, Figure 7, and Table 1). Intuitively, this finding is reasonable: population sequence data discretely sample a continuous process and reversion is marked as occurring at the first single time point it is observed. However, reversion of virus to WT may have occurred *prior* to this sampled time. Consequently, the estimates based on this approach should become more conservative as the frequency of sampling decreases. The modeling approach presented here offers one means of overcoming these issues by considering the process of reversion along a continuum.

This analysis provides a novel framework for developing a quantitative understanding of resistant variant evolutionary dynamics. The model enabled prediction of the median time-to-1% resistance for HCV subtypes 1a and 1b (11.0 months and 2.1 months, respectively) even though many patients solely had population sequence data available, which can only be used to determine reversion to 20% resistance. These model predictions (median time-to-1% reversion) were comparable to the median time-to-20% reversion as determined by a previous analysis using only population sequence data [14]. Significantly, these predictions suggest that if a patient does not achieve an SVR with telaprevir-based treatment, the viral population is likely to contain less than 1% resistant virus within a year following treatment failure. These findings provide additional quantitative information for patients with HCV infections and health care providers concerned about the ramifications of not achieving SVR with current treatment options.

## Methods

### Patient Population and Sequence Dataset

The dataset used for this analysis was previously reported by Sullivan et al. [14] and is briefly described here. Samples were obtained from 388 patients who had been enrolled in the Phase 3 telaprevir studies (ADVANCE [7], REALIZE [8], and ILLUMINATE [24]) and did not achieve an SVR. The Phase 3 studies evaluated either 8 or 12 week telaprevir treatment durations with 24 or 48 week durations of peg-IFN and RBV. Follow-up assessments monitored the retention of resistant variants for those patients who did not achieve an SVR.

### Determining Percent Resistance from Various Sequence Data Types

Population sequence analysis was performed with a minimum of 11× coverage of the NS3 protease, and a median of 4 time points per patient as described by Sullivan et al. [14]. In this analysis, a mutation for a given variant is coded as ‘not present’, ‘present as a polymorphism’, or ‘present and monomorphic,’ which quantitatively correspond to frequencies of <20%, between 20 and 80%, and ≥80%, respectively. As an alternative to population sequence analysis, clonal sequence analysis can be used to quantitate each variant with a theoretical 95% confidence limit of detection of ∼5% when 96 clones are sampled. For this analysis, clonal sequence data were obtained for a subset (n = 51) of samples for which population sequence data were already available. Clonal sequencing utilized the 9 KB amplicons used for the direct population sequence analysis. These amplicons were cloned into a TOPO PCR-XL® vector (Invitrogen) and transformed into electrocompetent *E. coli* as previously described [25]. Plates containing transformed *E. coli* were sent to Beckman Coulter Genomics (Danvers, MA) or Genewiz (Cambridge, MA) where 96 clones were selected for sequencing of the NS3 protease with a minimum of 3× coverage. If less than 50 clones contained inserts in a given sample, the process was repeated for that sample. A median of 2 time points and a median of 82.5 clones were available per patient with an interquartile range (IQR) of 70–91. From 32 genotype 1a patients, samples from 80 total visits were obtained with a median (IQR) of 79.5 (69.75–91.25) clones analyzed per visit. From 19 genotype 1b patients, samples from 54 total visits were obtained with a median of 84.0 (71.25–90.75) clones analyzed per visit.

### Modeling Resistance Dynamics

For patients that did not achieve an SVR, resistance evolutionary dynamics after treatment were modeled as a competition between the WT virus and telaprevir-resistant variants (an example from a hypothetical patient is shown in Figure 1). Under the assumption that in the absence of telaprevir, the WT virus is more fit than telaprevir-resistant variants [10], [14], [19], the target-cell limited viral dynamics were approximated using a logistic model for the WT virus, *V*:
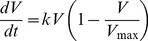
(1)Here *k* is the rate at which WT virus out-competes telaprevir-resistant variants. We assumed that *V* is the percent of the population that is WT virus; therefore *V*_max_ is 100%. Solving Equation 1 for *V* then yields:
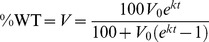
(2)Here *V_0_* is the amount of WT virus present at the beginning of resistance monitoring after the patient did not achieve an SVR and *t* is the relative time from the start of resistance monitoring. Equation 2 has been derived by Ganusov et al. [23] from more complex viral dynamic models under the limiting assumptions of (1) a variant initially present at low frequency (in this case, WT) and (2) a small mutation rate.

The percent resistance (i.e., quantifying the fraction of resistant variant) is simply:
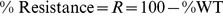
(3)

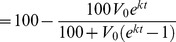
The dynamics in each individual patient were characterized via Equation 3 by specifying the parameters *V_0_* and *k*.

### Fitting the Model to Individual Patient Data

Qualitative and quantitative information on percent resistance (*R*) were obtained from population and clonal sequence data, respectively. Because clonal sequence data are quantitative, squared deviations between the model predictions and the percent resistance determined by clonal sequencing for a given sample were penalized. Statistically, this approach is equivalent to assuming that the clonal sequence errors are independently and identically distributed. Because population sequence data are qualitative, only model predictions that fell outside of the expected population sequence binned quantitative range (0–20%, 20–80%, and 80–100% resistance) were penalized. In this case, squared deviations were again penalized, with the deviation defined as the difference between the model prediction and the population sequence range extremum closest to the prediction. For example, if the expected range were 20–80% resistance, then a model prediction of 85% resistance at a given time point would have a deviation of 85%–80% = 5%. Similarly, if the expected range from a direct population sequence result were 20–80% resistance, then a model prediction of 10% resistance would have a deviation of 10%–20% = −10%.

The log_10_ values of the model parameters *V_0_* and *k* were determined by minimizing the sum of squared errors over all population and clonal sequence data points for a given patient. Computationally, the population sequence data points were handled efficiently as soft constraints, a technique used in advanced process control (see [26] and the references contained within). Assuming there are *n_p_* population sequence and *n_c_* clonal sequence data points, the optimization problem is then:
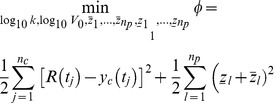
(4)

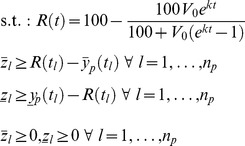
Here:

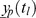
 is the lower bound of the population sequence constraint at time *t_l_*,

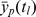
 is the upper bound of the population sequence constraint at time *t_l_*,

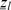
 is the soft constraint for the lower population sequence bound,

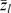
 is the soft constraint for the upper population sequence bound,

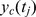
 is the clonal sequence measurement at time *t_j_*,
- *n_p_* is the number of population sequence data points, and
- *n_c_* is the number of clonal sequence data points.

For example, considering an expected range of 20–80% resistance with a model prediction of 85% resistance, the constraints in Equation 4 dictate that 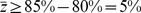 and 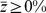, and since we minimize over 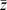,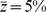. Similarly, the constraints in Equation 4 also dictate that 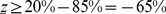 and 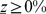, and since we minimize over 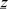,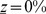.

### Fitting the Model across a Population of Patients

Data from patients that had both population and clonal sequence data were first fit using Equation 4. The parameters *V_0_* and *k* for the entire population were approximated using a log-normal distribution; this distribution was selected to enforce positivity of the parameters. The mean (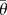) and covariance (Π) for this distribution (*θ*, consisting of both log_10_ *V_0_* and log_10_ *k*) were calculated from the subset of patients with both population and clonal sequence data. Finally, data for each individual patient in the population were fit using Equation 4 augmented with a term penalizing deviations of *θ* from its prior:
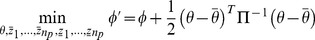
(5)s.t.: same constraints as Equation 4

Some exceptions were made to this strategy:

1. To maintain a conservative methodology, patients whose samples at the time of treatment failure had <20% resistant virus were assumed to have exactly 20% resistance at the time of failure.
2. If the sample collected at the final time point for a patient had 80–100% resistant virus as determined by population sequencing, virus in that patient was assumed never to revert to WT.
3. If the sample collected at the initial time point for a patient had ≥20% and <80% resistant virus as determined by population sequencing, only deviations of the log_10_ *k* parameter from its prior distribution were penalized. Deviations of the log_10_ *V_0_* parameter from its prior distribution were not penalized.

### Assessing the Model Predictions with the Experimental Assessment Dataset

Model predictions were compared to results of 52 samples generated by deep sequencing (DS) [19]. The limit of detection for this form of massively parallel sequencing was ∼1%. This dataset was compared against model results generated in this analysis if the population sequence result were ≤80% resistance and clonal sequence results were not available at a given time point (n = 37). Assessment of the model was performed using two methods: (1) Monte Carlo simulation and (2) an assessment of the null hypothesis that the model-predicted results were not different from the observed (actual) results. In both cases, a quantitative result at some time *t* after treatment failure was obtained from the DS dataset. The modeled patient-specific parameters *V_0_*, *k*, and *t* were used to solve Equation 3 for *R*. Given a limit of sensitivity for the DS assay of 1%, if the observed (actual) result were below the assay LOD, the actual % resistance was imputed as 0%.

For the Monte Carol simulation, a sum of squared errors was calculated from the difference between the actual and model predicted % resistance. Monte Carlo simulations assumed a uniform distribution within the measured population sequence range. 10,000 replicate datasets were sampled to generate a distribution of errors. The significance of the model prediction was determined by calculating the percentage of errors in this distribution that were less than or equal to the observed error.

To assess the null hypothesis that the modeled results were not different from the actual results, the difference between the actual and predicted result was determined for each sample, and the null hypothesis that the mean of this difference equaled 0 was tested. Because the resultant differences were not normally distributed, the absolute differences were first arcsine square root transformed, but the original sign of the difference was retained. Parametric (*t*-test) and non-parametric (rank-sign) tests were performed on the resultant dataset with α set to 0.05 for each test.

### Estimating Median Reversion Times for the Patient Population

Once parameters for all patient samples were estimated, the modeled time required for virus from samples of each patient to reach a specific percent resistance was calculated by setting the % resistance (*R*) in Equation 3 and solving for *t* (specific time-to-x% denoted by *τ_x%_*). Kaplan-Meier curves were constructed to determine statistics for the population *τ_x%_*. Patients who are modeled to retain resistance throughout their post-treatment follow up were considered right censored in the Kaplan-Meier analysis, with the time of the last observation in the population sequence analysis dataset relative to the time after treatment failure used as the time of the censoring event. To determine the effect of covariates on time-to-1% reversion, Cox proportional hazards models were applied across relevant covariates on the Kaplan-Meier analysis, with this analysis performed separately for genotypes 1a and 1b and with a null hypothesis of no effect.

### Numerical Implementation

Population and clonal sequence data were queried using a custom Oracle database with Perl scripts. R (v. 2.15.0) was used to convert these numerical values into a format readable by Octave, which was in turn used to estimate model parameters using the optimization routine sqp.m. R was used to generate figures and calculate Kaplan-Meier statistics using the survival library. Kaplan-Meier statistics were confirmed by independent generation with JMP statistical software (SAS Institute, v. 8.0.1).

### Data Availability

To ensure patient confidentiality, an anonymized dataset containing a summatry of the raw data underlying these analyses has been created and is available upon request to globalmedinfo@vrtx.com.

## Supporting Information

Figure S1 **Number of patients by HCV genotype with a given amount of population and clonal sequence data points.** Results are for patients with (A) genotype 1a HCV and (B) genotype 1b HCV. The “Sum” column shows the total number of patients with a given number of clonal sequence points (the row sum).
(EPS)

Figure S2 **Forest plot of covariate effect estimates on the time it takes to revert to 1% resistance.** Kaplan Meier analysis was used to describe the time it takes for patients to revert to 1% resistance (Figure 6). The figure displays the effect estimates and 95% CI for those effects based on application of Cox proportional hazard models to the Kaplan Meier analyses.
(EPS)

Figure S3 **Monte Carlo model predictions generated by importance sampling for representative patients with genotype 1a HCV.** Representative Monte Carlo simulations for three patients with genotype 1a HCV. Gray lines represent the model predictions from the 1000 Monte Carlo parameter sets generated by importance sampling. Red lines represent the median of those 1000 simulations. Solid points represent the clonal sequence data. Error bars show the range for population sequence results.
(EPS)

Figure S4 **Variability in the Kaplan-Meier analysis due to uncertainty in the parameter estimates for individual patients.** Kaplan-Meier analysis for patients with (A) genotype 1a and (B) genotype 1b HCV. Solid and dashed lines represent the median results and 95% confidence intervals of 1000 independent Kaplan-Meier analyses. Caret marks (∧) denote patients who did not revert to WT during the follow-up period. For clarity, these patients are explicitly denoted on the population sequence (“Pop. Seq.”) resistance curve only.
(EPS)

Text S1 **Supporting information related to quantifying uncertainty in Kaplan Meier point estimates.**
(DOCX)
