# Supplementary material for: Modeling Viral Evolutionary Dynamics after Telaprevir-Based Treatment
Source: PLoS Comput Biol. 2014 Aug 7;10(8):e1003772. doi: 10.1371/journal.pcbi.1003772 (PMC4138032; doi:10.1371/journal.pcbi.1003772)
Supplement: Text S1 — Supporting information related to quantifying uncertainty in Kaplan Meier point estimates. (DOCX) [file pcbi.1003772.s005.docx]

# Supplemental Results

## Quantifying uncertainty in the Kaplan-Meier analyses

The population statistics for reversion time determined by the Kaplan-Meier analysis did not account for variability due to uncertainty in the parameter estimates of individual patients. Monte Carlo simulation was used to determine how this uncertainty affects the Kaplan-Meier analysis. Briefly, importance sampling was used to generate 1000 parameter sets for each patient using the approach of Brown and Sethna and Gutenkunst et al. [1,2]. Plots of the resulting model predictions for representative patients are shown in Supplemental Figure 3. Patients with only population sequence data (e.g. “Patient A” and “Patient B”) exhibited greater uncertainty in their reversion dynamics than patients for which clonal sequence data was available (e.g. “Patient C”).

Using the Monte Carlo sampled data for all patients, 1000 independent Kaplan-Meier analyses were performed and the median and 95% confidence intervals for the median response were calculated. The uncertainty in the parameter estimates for individual patients contributes minimal variability to the median Kaplan-Meier curve for the reversion dynamics (Figure S4). Whereas relatively large variability was observed in the reversion time from individual patients, particularly those with only population sequence data, the Monte Carlo simulation of the Kaplan-Meier analyses suggested that variability from individual patients averages out across the population with respect to the median reversion dynamics.

**References**

1. Brown KS, Sethna JP (2003) Statistical mechanical approaches to models with many poorly known parameters. Phys Rev E 68: 021904.

2. Gutenkunst RN, Waterfall JJ, Casey FP, Brown KS, Myers CR, et al. (2007) Universally Sloppy Parameter Sensitivities in Systems Biology Models. PLoS Comput Biol 3: e189.
